# Seafarer Fatigue: a qualitative study of customer-facing staff working on UK ferries

**DOI:** 10.3389/frsle.2026.1906714

**Published:** 2026-08-21

**Authors:** Sally Maynard, Wendy Jones, Adam Asmal, Anna Anund, Andrew Pooley, Ashleigh Filtness

**Affiliations:** 1 School of Design and Creative Arts, Loughborough University Transport Safety Research Center (TSRC), Loughborough University, Loughborough, United Kingdom; 2 The Swedish National Road and Transport Research Institute (VTI), Linköping, Sweden

**Keywords:** customer-facing seafaring staff, fatigue, risk factors, self-devised countermeasures, sleepiness

## Abstract

**Introduction:**

This study explores the experiences and opinions of customer-facing seafaring staff on the subject of fatigue and how it affects their work and interactions with passengers. This group is of particular interest as were there to be an emergency at sea it is those in customer-facing roles that would take responsibility for passenger safety.

**Methods:**

Data was collected from focus groups with customer-facing staff (n = 45 participants across nine discussions).

**Results:**

Fatigue was seen to be a problem, with all focus group participants having experienced sleepiness while at work and being able to detect fatigue in others. Factors identified as contributors to fatigue included: commuting, external factors such as the weather and onboard noise and shift and roster patterns. A variety of informal, self-devised (that is, not directed by a manager) countermeasures were being employed by the focus group participants, with caffeine in the form of coffee being the most common. Participating seafarers suggested that adrenaline carries them through emergency situations so that fatigue experienced after an incident was more of a concern to them than that encountered during it.

**Discussion:**

The research represents one of the first qualitative studies to subjectively investigate fatigue amongst customer-facing staff on UK ferries, understanding their lived experience is a first step toward mitigating sleep related risks for this job role. Recommendations for ferry operating companies are made.

## 1 Introduction

Inherently global in its nature, the shipping industry is complex and operates on a 24-hour basis. The intensive nature of some shipping operations means that the seafarers who work in the industry may be subject to long and/or irregular work hours because the shipping companies employing seafarers (used here to indicate anyone undertaking paid employment onboard a sea vessel) rely on unconventional working patterns to accommodate the demands of the industry and to adhere to the complex relevant working regulations (HORIZON, 2012).

This research is focused on the ferry sector, a key segment of the shipping industry. It specifically focused on the roll-on roll-off passenger (Ro-Pax) ferries operating in the waters around the UK. The Ro-Pax ferries carrying passengers from port to port undertake approximately 37,000 crossings each year, with almost 30 million passengers [Department for Transport (DFT), 2024]. The shipping companies operating these ferries provide a commercial service and establish route connections between ports where they believe they can be profitable. At the time of data collection thirteen companies were operating ferries around the UK. To deliver this service, each company will design a range of shift and roster patterns to enable them to meet the demands of their routes. Shift refers to the number of hours of work and rest within a 24 h period e.g. 12 h of work followed by 12 h of rest. Roster refers to the number of days in a particular period which contain work shifts e.g. two weeks on followed by one week off. The crew of some vessels travel home every day after working their required shift hours (as employees would in most non-maritime workplaces) and others sleep onboard the vessel (HORIZON, 2012; Behavioural Insights Team and Transport Research Laboratory, 2023). These working arrangements mean that seafarers' working hours may deviate from more usual patterns and instead be subject to shift work, variable rosters, and irregular watch schedules.

As with all shift workers, seafarers are at increased risk of sleepiness. Within the ferry sector the term fatigue is commonly used to encompass the experience of both task related fatigue (an experience of tiredness due to an activity being undertaken which increases over time) and sleepiness (an experience of tiredness due to insufficient sleep or circadian influence due to time of day). However, within the industry it is common to use the term fatigue when referring to either fatigue or sleepiness. Therefore, the term fatigue will be used in the current work as it is more familiar for participants and their employers. Fatigue is a recognized risk factor for incidents in seafaring. High-profile fatigue-related incidents in the last 30–40 years have involved tankships including the Exxon Valdez and the Eagle Otome, naval vessels such as the USS Fitzgerald and the USS John S. McCain and passenger vessels such as the Star Princess (Shattuck et al., 2023). Investigations into these found common underlying fatigue triggers including long hours, shift work, missed sleep, and nighttime operations: all of these can be present throughout the maritime sector, indicating the important need to manage sleep related safety risks for this industry. Fatigue led to crew being cognitively impaired, making errors of judgement and unable to communicate effectively. In some cases, multiple crew members were fatigued at the same time, compounding the difficulties. Similar incidents have occurred in the ferry sector, a recent example being the Alfred, a passenger ferry which ran aground in Scotland in 2022, causing 41 injuries. Fatigue was found to be the primary cause of the incident, with the master falling asleep for around 70 seconds whilst navigating the vessel close to shore (MAIB, 2024).

The impact of fatigue on seafarers has previously been investigated. For example, the effects of sleepiness on the cognitive performance of maritime watch keepers under different watch patterns through simulation studies have been investigated by HORIZON (2012). A follow-on study, MARTHA (2016), involved a sample of volunteer seafarers in the naturalistic setting of work onboard their vessels to explore levels of sleepiness and the psychosocial issues associated with long term fatigue and motivation. Both projects confirmed fatigue to be an important issue for seafarers and managers, with safety, long-term physical and mental health implications. It was also found that the working patterns led to a high probability of reduced sleep and of increased fatigue, with an ensuing accident risk (HORIZON, 2012; MARTHA, 2016). Furthermore, a systematic literature review undertaken by Dohrmann and Leppin (2017) to detect, analyse and assess current thinking around the subject of seafarer fatigue found a need for further investigation of realistic countermeasures and a wider range of factors and interactions between factors.

There is a dearth of research investigating issues of fatigue specifically within the ferry operating sector with a literature review finding very little recent ferry-related literature (Behavioural Insights Team and Transport Research Laboratory, 2023). Some notable exceptions include Dohrmann et al. (2019) questionnaire survey which showed that work-family conflict was fond to be positively associated with fatigue, particularly in terms of lack of energy, physical discomfort, lack of motivation and sleepiness. Additional support from supervisors was related to a reduction in lack of energy, physical exhaustion and lack of motivation. The authors recommend that shipping companies should consider introducing initiatives which reduce conflicts between family life and work obligations. Dohrmann et al. (2020) aimed to investigate work stress and fatigue among ferry ship employees and found support for sleep satisfaction in a mediating role. The authors suggest that ferry operators should implement sleep supporting initiatives, for instance in the form of engaging employees in duty roster-planning in combination with comfortable beds that support intra- and inter-shift recovery. However, there was limited focus on customer-facing staff within previous work.

Where research does exist it often targets those operating the vessel, however, should a major incident occur at sea on a Ro-Pax vessel, it is the customer-facing staff who are responsible for safe passenger care and evacuation. This responsibility incorporates the risk of injury and loss of life, and such duties may therefore affect the experiences of customer-facing staff particularly with regard to their feelings of fatigue. To our knowledge, the current work is the first to investigate the impact of fatigue for customer-facing seafarers (e.g. catering, housekeeping and shop workers). Such staff are usually designated onboard sales and services (OSS) staff. In addition, the invitation to participate was extended to some able-bodied seafarers (ABs), since their role is also partly customer-facing when they are taking the lead in loading and unloading passenger vehicles.

The research aims underpinning the focus groups were as follows:

- To investigate the impact and potential consequences of fatigue for customer-facing seafarers
- To understand the effect on fatigue of the particular role responsibilities for passengers in an emergency
- To determine the most important factors causing and exacerbating fatigue for customer-facing seafarers

## 2 Methods

A qualitative approach was used because it helps understand attitudes and elicit industry-specific insights around complex issues. Qualitative methods can also provide a more holistic understanding and, by helping identify constructs beyond the commonly known (Stiegemeier et al., 2022), also contribute to the current body of literature. Specifically, focus groups were used in order to bring participants together to explore the topic of fatigue within a social context. When exploring an everyday work occurrence, it was hoped that the group setting would elicit greater personal insights through conversation about shared experience than would have been possible from individual data collection by interviews. Where possible discussion groups were held on a vessel in order to create *in situ* experience when being asked to consider fatigue. The discussion was designed to identify the participants' views on the causes and consequences of their fatigue experience at work.

The design used in the focus groups was partially informed by previous research into fatigue which utilized focus groups with bus drivers (Maynard et al., 2021) and construction workers (Maynard et al., 2020). Each focus group followed the same format. The questions took into consideration fatigue in the context of both day-to-day experience and in emergency situations. Focus group participants were also asked to describe events at different stages of a voyage.

In accordance with Loughborough University's code of practice for research, ethical clearance was sought and gained via the relevant procedures.

### 2.1 Participants/recruitment

In terms of job role, the sampling strategy was purposive because all participants were required to have customer facing roles; however, within that restriction convenience sampling was undertaken in line with the practical constraints of hosting the groups.

The UK Department for Transport (DfT) is responsible for maritime transport policy, and as part of the DfT commitment to evidence-based policy-making they regularly fund research activity across all transport modes, including the work reported here. In addition to financial support the DfT also introduced contacts at companies operating ferries to the research team. Three companies operating Ro-Pax ferries agreed that focus group discussions could be held on their vessels and gave researchers permission to recruit volunteers from their workforce via their managers. Up to eight seafarers working in a customer-facing role were invited to each focus groups. The groups took place on the vessel on which the participants were working in a room suitable in size and privacy.

To widen participation opportunity two online discussion options were provided. These were advertised via the relevant trade unions, allowing the participation of customer-facing staff working at ferry operating companies which had not agreed to host the groups. Two online groups were hosted, however, only one participant attended the second group. As shown by Table 1, nine focus groups were completed (seven onboard (face-to-face) and two online); a total of 45 seafarers participated in the discussions. The sampling process was designed to collect data relevant to the sector, as such there was a target to engage with participants from at least three (23%) of the 13 potential ferry operating companies. Demographic information about participants (other than job role) was not collected.

**Table 1:** Summary of focus group participants.

Focus group	Job role	Number of participants	Face-to- face/online	Ferry operating company	Vessel
FG1	OSS	8	Face-to-face	A^2^	1
FG2	OSS	8		A^2^	1
FG3	OSS	8		B^3^	2
FG4	AB	2		C^3^	3
FG5	AB	2		C^3^	3
FG6	AB^1^	5		C^3^	4
FG7	OSS	7		B^3^	5
FG8	OSS	4	Online	N/A	N/A
FG/interview	OSS	1		N/A	N/A
**Total**		**45**		**3**	**5**

^1^ One bosun attended ^2^ Seafarers travel home after work ^3^ Seafarers sleep on board during rostered work hours.

### 2.2 Procedure

Prior to each focus group, participants were sent an information sheet and returned completed informed consent and demographic forms. Table 2 provides an overview of the structure of focus groups, topics and example questions. The focus group protocol was developed iteratively by the research team to ensure the groups followed the same format and promoted reliability in data collection.

**Table 2:** Overview of the semi-structured focus group protocol.

Topic	Content/example question(s)
Introduction	Welcome, overview of the project, ethics and confidentiality
Does fatigue occur?	“If I say “fatigue,” what do you think about?”
Understanding fatigue in workplace culture and personal experiences	“Have you ever experienced fatigue at work? Can you share a situation where you or someone you know felt tired at work?” “Can you give examples of situations that commonly lead to fatigue during your work routine?” “What would you do if you felt fatigued or sleepy at work?”
Exploring the impact of shift work on Fatigue	“How do external factors, such as environmental conditions or external pressures, contribute to your fatigue? How might these factors vary across different work stages?”
Exploring the impact of fatigue in daily work and in high-pressure situations	“Do different stages of a voyage impact your fatigue levels? Are there specific stages that you find more challenging?” “Do you have any responsibilities when faced with a high-pressure emergency situation on the vessel?”

The participating seafarers were encouraged to treat the experience as an informal discussion with a focus on fatigue and how it is managed at work, and to express their honest opinions. Focus group participants were urged to talk to each other and to the researchers during the discussion and thereby share experience. It was emphasized to participants that all information they provided would be confidential, with no individuals or ferry operating companies being identified.

The seven face-to-face discussions took place on the ferry on which the participating seafarers were currently employed; they occurred in a private room where the conversation could not be overheard by anyone outside, and no managers were present. Each face-to-face focus group was facilitated by two researchers working together; they were taken from a group of four available researchers. The maritime industry tends to have a hierarchical culture and many seafarers are ex-military which likely contributes to this workplace culture. A deliberate decision was taken for researchers to be presented as “out of group” with an intent to create a friendly and non-judgmental feel to conversation. None of the research team had previous connections to the maritime industry.

Online discussions took place using MS Teams. The discussion undertaken with a single attending participant followed the same questions as the focus groups. Both online focus groups were facilitated by the same one researcher, who was also part of the group of four facilitating the face-to-face groups. The participants of the online focus groups did not know each other prior to participation, whereas those in the face-to-face group were work colleagues. Effort was made in the online group to initially put participants at ease and introduce them to each other, and the tone set by the researcher supported collaborative conversation between participants. The focus group data collection took place during March and April 2024 and an incentive was offered to the participants (£20 per participant) to compensate them for their time.

### 2.3 Analysis

The recordings from the focus groups were transcribed verbatim, de-identified and analyzed using a thematic approach (Braun and Clarke, 2006) via NVivo (ver. 14.0) software allowing themes to develop both from the research questions and from the narratives of the focus group participants. The dialogue from each focus group was coded in two stages of qualitative thematic data analysis: (1) open coding and (2) axial coding (Saldana, 2009). Given the size and richness of the dataset, these two stages of analysis created a robust process, ensuring all data were captured and considered during analysis. The themes were discussed and devised by two researchers who had both been part of the data collection team. Prior to data collection activities the two researchers immersed themselves in the ferry industry through extensive reading of both scientific and gray literature related to the sector. They also undertook a “ride along” experience whereby the were given open access to a vessel undertaking two crossings in order to observe the day-to-day working experience without collecting data. Both coding researchers were experienced specialists in qualitative research. The researcher who led the analysis had recently conducted similar studies (focus groups and interviews) investigating experiences of fatigue in bus drivers and tunellers. Much of their work was focused on transport safety, including work with train drivers, network rail track workers and bus network controllers. The second coder had a background in construction and education research as well as being an experienced occupational health care professional. In both cases, the researcher experiences may have influenced the analysis, particularly in terms of understanding and appreciating the challenges faced by the participants.

Both researchers had been involved in data collection on board the vessels where the focus group participants worked. Their own experiences here enhanced their understanding of the topics discussed in the focus groups and provided background context, but again, may have influenced their interpretation of the data.

To counter the risk of bias, the researchers carefully checked the conclusions reached against the focus groups transcripts to ensure that they were rooted in the data, rather than in their own personal experiences. The first stage consisted of open coding, which identified units of information that addressed the research aims. This was a bottom-up approach to obtain first impressions from the transcripts and was led by the views and narrative of the focus group participants. At this stage researchers reflected on the potential for online groups to yield different data compared to face-to-face groups; no substantive differences were noted so data from both types of data collection were considered together. The previous knowledge of the research team and the research questions were also taken into consideration. During this stage, the two researchers read through the data and started to create tentative labels for sections that summarized the ideas arising from the data. The two researchers separately coded the transcript from the first completed focus group in this way. The researchers then met to undertake inter-researcher triangulation whereby they compared their analysis and subsequently refined and agreed the final set of codes. An audit trail was maintained of the individual coding for this focus group, accessible to the other authors of this work.

In the second stage (axial coding), relationships were identified among the open codes; these resonated with pre-established constructs derived from the context of the work of customer-facing seafarers and research relating to fatigue so that the core themes could be identified. This axial coding was a critical step for relating categories and concepts with one another and refining the categories. The codes, which had been drawn from the data by the two researchers during their initial analysis, were compared and contrasted in order to rationalize them. Once the researchers had ensured that all of the topics raised were covered by their new codified group, a set of four codes (or themes) were defined; these were then used to analyse the remainder of the focus groups, with each group being analyzed by one of the two researchers who attended all of the sessions.

As can be seen in Table 1, six of the groups (including the focus group with one attendee) involved OSS staff. There were 36 participants with OSS roles; their job titles included steward, mess man, chief cook, cabin assistant and cleaner, amongst others. In addition, three focus groups were held with the participation of a total of eight ABs and one bosun. These groups were included since ABs also have responsibilities for passenger safety, particularly in emergencies, and have some day-to-day interaction with passengers albeit more limited than OSS. It was therefore of value to investigate the opinions of seafarers working in the AB role. Outcome of analysis was not subject to member checking.

## 3 Findings

The findings from the focus groups center around four main themes: causes of fatigue; recognizing and reporting fatigue; experiences during emergency incidents and countermeasures to fatigue. All the higher level themes were raised in all nine focus groups.

It is recognized that the experience of fatigue is multifaceted, encompassing pressures from both the sleepiness related to human biology and task-related fatigue, therefore, a broad scope for coding was taken. This might include sleepiness due to insufficient sleep and/or time of day but may also refer to task-related fatigue due to the nature of work as a seafarer. This accorded with the responses to the introductory question posed to all focus group participants asking them to describe their understanding of the concept of “fatigue.

“The responses included words and phrases such as “*tiredness*,” “*exhaustion*” and “*lack of concentration*.”

For example,

“…* it'll be something along those lines, of tired, exhausted, burnt out.”* Views of fatigue also encompassed both mental and physical fatigue, for example, “*fatigue to me is physical, emotional, mental tiredness.”*

### 3.1 Causes of fatigue

The multifaceted nature of fatigue means that multiple factors can be causes of fatigue. Within the broad theme of causes, six sub-themes have been created representing the focus group participants' lived experience. These are: commuting (both for those living on board and those sleeping at home); external factors such as the weather and vibration and noise on the ship; seasickness; shift patterns and particularly the first shift on a roster; shift swapping and overrunning; and roster patterns.

#### 3.1.1 Commuting (seven focus groups)

Commuting to the workplace was evident as a cause of fatigue for all seafarers but featured more prominently in group discussions for those who sleep at home, likely because they travel more regularly. For these participating seafarers, the commuting necessarily involved traveling daily. This might be a quick journey,

“*I walk to the ship. I live ten minutes outside of port,”* or it might be by car or public transport. This would have an effect on focus group participants' opportunity to sleep during their time off work, “*so even though we've got our 12 hours [between shifts], we don't because we're traveling at least a good hour and a half sometimes."*

For those living on board the commuting journey occurred less frequently, but when it did, for many the journey to the vessel was long, with focus group participants discussing colleagues being

“*dotted around the whole country.”*

This is particularly the case for those living outside the UK as their journey might be counted in days.

Another concern related to commuting was the ship running behind its planned schedule at the beginning of the shift. This would mean that individuals would be unable to join the vessel at the expected time which could in turn affect their plans for sleep. Particularly for a night shift, this would mean reporting straight for work when a seafarer had been up for a longer period than they might otherwise have been. As one focus group participant said,

“*If the ship's late … you join and you're on nights, you're straight into it. You'll have no time for rest.”*

Although not explicitly discussed, this would also have an impact on the opposite number who might then disembark and start their commute while fatigued.

#### 3.1.2 External factors (nine focus groups)

A range of external factors outside the control of participating seafarers were identified as being contributors to fatigue. The first was lack of crew, either due to sickness amongst workmates, or colleagues having left because of various reasons including unhappiness with the company or job role. Having insufficient workers will necessarily lead to an increase in the workload expected of those who are present, although this is mitigated somewhat by the legal requirement to have a minimum number of crew on board the ferry before it can sail. This legal requirement may lead to certain seafarers being required to work extra shifts to cover for missing colleagues, as to meet safety requirements for vessel operation there are tight regulations around job roles required. While the intention is to ensure safe sailing, requesting colleagues to remain on shift in order to meet minimum staffing requirements introduces a risk of fatigue due to the potential increase in their number of work hours or days.

Another key concern expressed by the focus group participants who live on board was the general noise associated with the operation of the ferry. This included the Tannoy system and any alarms which may be broadcast at all times of day and night, some of which may require seafarers to leave their cabin. Another source of noise is the docking of the vessel which is likely to be combined with the vibration of the ship's thrusters which rattles their possessions within the cabin. Heat on board the vessel may also be a cause of tiredness and may also affect the seafarer's ability to carry out their work.

Noise can also emanate from colleagues living on board, with seafarers attempting to sleep at a variety of times across the day and night. Sharing a cabin may also be a source of disturbance to sleep, although attempts are made to minimize the possibility by assigning those on the same shift, or those on directly opposing shifts to a cabin so they are both on rest at the same time or at opposite times.

The weather is a particular concern for seafarers, with the vessel being subject to this as an uncontrollable force. Rough seas make the work itself harder and mean that it can be difficult to sleep on board, for example,

“…* sometimes when the weather is bad and you don't sleep, well then you are more physically fatigued”* and “*when it's rough weather, I seem to be more tired”*. This included being physically tired: “*you have use more muscles … when it's rough because you have to stabilize yourself. Whether that's your torso, your abs, whether you have to use your hands, hold on to stuff. So, obviously it's more tiring, obviously to be on the rough seas*.”

Another problem connected with rough weather was the effect on the ferry schedule, with journeys likely taking longer than usual. This may result in longer shifts or changes in start and end times of working periods, resulting in feelings of fatigue.

The passengers themselves can also be a source of tiredness for the focus group participants; this may either be due to large numbers or demanding passengers. For example,

“*another thing that I'd say is, adds a lot of stress and tiredness is angry … abusive passengers because we get a lot of that as well … once you get them off the ship all of a sudden, you … drop right down and it becomes real tired, really tired really quickly.”*

Notably, rough weather may cause the passengers to be more demanding and may subsequently add to the workload of the participating seafarers.

The focus group participants confirmed that seasickness (three focus groups) can be a concern for them, particularly on rough days. It was also dependent on which vessels they were working on, reportedly because of some ships vibrating to a greater extent than others. Those who take medication for seasickness also noted the possibility that it can cause them to feel drowsy. For example:

“*I do find that it's also the sea sickness tablets that's meant to help you make you fall asleep as well, it just tires you out even more.”*

The seasickness itself can also render the seafarers so ill that they are unable to work. However, there are also occasions when the seafarers noted carrying on with their work,

“*you'll run off, be sick, do what you need to do, then you're back working. You can't think about it … you're just working*.”

This was thought to be a drain on the focus group participants, meaning they are losing nutrients and not having time to drink thereby contributing to their feelings of fatigue.

#### 3.1.3 Shift patterns (nine focus groups)

A clear perceived cause of fatigue for the participating seafarers is shift working, and it was usually the first response from them when they were asked about causes. It is thought to be a reason for participating seafarers receiving insufficient sleep, with many believing eight hours to be the accepted requirement for sufficient sleep. The seafarers involved in this research worked a variety of shift patterns, some of which might be split and others which are straight shifts usually lasting 12 h.

Split shifts were reported as being potential barriers to sleep, so those working six hours on/six hours off would not be able to have a useful period asleep and, as noted by one focus group participant,

“*getting four hours twice a day is not the same as getting eight hours uninterrupted at all.”*

Straight shifts (usually 12 h work with 12 h of rest) were preferred by some of the focus group participants, mainly due to the longer period of rest. It should be noted that breaks during work would customarily be scheduled into this kind of shift. Some of the participants were working 13-h shifts (with three unpaid 20-min breaks) which they found tiring, particularly when the shift over-ran and they were working for a longer period.

Night shifts were generally considered to be the most difficult for the focus group participants, with one commenting,

“*the only time I'm really tired is when I'm on nights. I think my whole body goes all over the place.”*

It was also suggested that it took a longer period to recover from a week of nights than from a week of days.

The changeover between night and day shifts was reported as a particular source of fatigue, for example,

“*it's constantly changing, one week you're early, the next week you're late, or it could even be the same week … it does … take its toll.”*

Furthermore, the frequent changes in daily start time for those who slept at home were thought to disrupt the body clock, which caused fatigue and were hard to become accustomed to. Variations in shift start patterns were generally attributed to the times when the ship was in port.

Constant alterations in the participating seafarers' shift patterns were also discussed, with changes being due to external factors such as the weather and the tides. This meant that focus group participants believed that they could not arrange events or appointments outside of work and that they could not “*really get into a routine because it's always changing.”*

#### 3.1.4 Swapping shifts (eight focus groups)

The main reason for swapping was due to staff absence, with staff being recalled to the ship during their time off. This could happen at any time, with staff being required to return to work sometimes at very short notice. It was suggested that those making the requests do not always take account of participants' rest time, although those seafarers who are asked can refuse to return.

There were some arrangements to allow the participating seafarers to voluntarily swap shifts, although this had to be approved formally through the operator and usually in good time before the swap would happen. Some of the focus group participants seemed reluctant to ask to swap, and others suggested it would need to be for a very good reason.

#### 3.1.5 Overrunning shifts

As might be expected, overrunning shifts are a concern to the focus group participants in terms of causing fatigue. These are generally due to the vessel being delayed because of inclement weather or other factors. It may involve the seafarers working a shift longer than it should be or beginning at a different time from when they had planned.

If the vessel is severely delayed over several days, the effect can be to change the shift pattern of the staff. As noted by one of the focus group participants,

“*all of a sudden you've gone from working your normal day shift to almost flipping and being on a night shift because you're that late.”*

The master might mitigate for this by keeping the vessel in the port.

#### 3.1.6 Roster pattern (nine focus groups)

The roster patterns of the participating seafarers can vary across different operators, vessels and across job roles; these were cited as a cause of fatigue by the participants. When asked about the effects of the two-week roster, one focus group participant said:

“*I think it's mainly the last few days before the end … because it's just that getting up at 5am every morning, and sometimes I go to bed at 9 or 10, and if you don't sleep well … say you didn't sleep well for a few days in the week, at the end, you're struggling to get up and wake up.”*

It was suggested during the focus groups that particular roster patterns may contribute to fatigue. For example, one participant said that the roster can lead to them working

“*two weeks compressed into one week”*. As a result, they then need that week off to recover, “*so basically three days, four days out of that you're just winding down, catching up with the world.”*

Another focus group participant also noted that even rest periods at home may not allow complete respite.

#### 3.1.7 Training for emergencies (three focus groups)

Drills on board the vessel are an essential part of seafarer training to ensure maritime safety; they are intended to refresh basic safety training and add an element of reality of working as part of a team on board. All the staff on a ship are assigned a muster number, which signifies their role during an emergency and the drill is intended as a practice for such an emergency.

Notably, the drills may take place at any time of the day or night and therefore this will necessarily be during the rest periods of several seafarers on board the ship. Generally, participants are not compensated for this time with further rest periods, although in some instances attempts are made to do so. Therefore, drills, whilst being of utmost importance in the safe operation of the ship, may also compromise the rest and sleep time of the staff on board and lead to safety concerns related to fatigue.

### 3.2 Recognizing and reporting fatigue

#### 3.2.1 Detecting fatigue in others (seven focus groups)

Most of the focus groups took place on board a vessel which meant that the participating seafarers were accustomed to working together on that same ferry. This brought about feelings of working within a regular team, with one focus group participant remarking “*it's like [a] second family for us”*. The focus group participants suggested that being familiar with their colleagues meant that they were able to detect fatigue in them, for example, “*when the heads are like nodding dogs”* with a general understanding that fellow workers are likely to be especially tired on their first shift of a rotation. As a result of such recognition, it was suggested that familiar teams would step in and help others, perhaps even allowing them to take a break.

#### 3.2.2 Training and advice (nine focus groups)

When asked whether they had been provided with any training on how to deal with fatigue, most of the participants were not aware of any such training. Although it was acknowledged that the trade union has widely recognized interests in the potential for fatigue amongst their members, it was clear that participants were generally proactive in seeking out information about fatigue rather than the companies providing it. The point was also made that, if the operator provides information about fatigue they should “*on the same hand respect it if you are suffering with that”*. The implication from the participants was that they do not.

#### 3.2.3 Reporting fatigue (nine focus groups)

The picture was varied regarding feeling able to report fatigue to someone else when at work. Some of the participants felt they would not be taken seriously and that they would be required to carry on working regardless,

“*I feel like you'd be laughed at”* and “*you'd get the sack.”*

Others suggested that the tiredness would be considered to be the fault of the individual who was allowing their life outside of work to impinge on their work time.

In contrast, for one group of ABs there was a general feeling of responsibility to report their fatigue. For example, one AB said,

‘*You've got to say to a bosun, “I need to have a lie down*”.'

For these participants the consequences of reporting were not feared, and they believed that they would be allowed some time off to rest. For example, “*I think if you actually went up to your senior and said*,

“*Look, I really need a break”, they would do it for you.”*

It therefore seems that the organizational culture around reporting fatigue for seafarers is variable across job roles as well as amongst operators and even across vessels.

### 3.3 Experiences during emergency incidents

#### 3.3.1 Incidents (nine focus groups)

Most incidents which the participants had encountered were of a medical nature, including passengers who had died or been injured on board the ship. There were well-understood protocols for such incidents; this included senior OSS staff being first aid trained (in addition to the master) and asking for help from medical professionals traveling on board where appropriate.

Another type of incident being met by the participants was fire, which seemed to be a routine incident that was anticipated, with well-understood actions to be taken, for example,

“*I've had a fire … we just had to get all the passengers away … but the crew put it out in the end.”*

There were other, more serious, incidents discussed, and the participants noted that there was no particular stage of the voyage at which incidents would be more or less fatiguing.

The participants were clearly very aware of their responsibilities in an emergency situation, describing it as being on call all of the time, including during rest periods in the case of those who live on board the vessel. This could lead to

“*the anxiety of worrying about something happening or something might happen.”*

As noted by one participant:

“*my muster duty would be to go down the MES chute first to help passengers … as they come down it. So that's a hell of a responsibility knowing that I'm responsible for everybody coming down that chute.”*

The participants agreed that some incidents had an effect on them, including on their feelings of fatigue as well as their mental health. The general agreement was that their adrenaline would hit them at the time of the incident enabling them to carry out their responsibilities as practiced during the drills, even if they were tired. However, it would be afterwards that they would feel fatigued. For example, the participant who said,

“*you're definitely on a rush of like you just get on with it and do what you have to do, but you definitely feel it afterwards … once it dies down.”*

The experiences of care given to participants after an incident were variable. The reaction and facilities offered by different operators were also inconsistent. For example, a participant discussed finding a deceased person in a cabin and commented:

“*we were asked if we wanted counseling, that was it, and we just had to get on with our jobs … because this was at 8 o'clock in the morning. We weren't asked if we wanted to come off the ship or be replaced”*.

A second participant noted a colleague not receiving any time off work after a similar incident involving a co-worker. A third participating seafarer discussed their workmate being involved in a medical emergency as follows:

“*He couldn't stay awake. But … the company … did not say … “Do you need an extra sleep? Do you need a time off?”.”*

The group of participants who had described working in a close-knit team suggested that this would be an advantage in the aftermath of an incident, with colleagues being able to help the affected workmate,

“*we're like a second family on here … everybody would come … to help out with whatever situation you were in.”*

There was some agreement that fatigue can lead to incidents, for example,

“*that's where generally all mistakes are made when people are tired … that's what it comes down to – a lot of it comes down to fatigue.”*

### 3.4 Countermeasures to fatigue

There was a wide variety of personal strategies for mitigating fatigue risk noted by the participants, with the most common being coffee, closely followed by energy drinks. As one participant said,

“*the shift runs on coffee.”*

Comments were made that energy drinks are more popular amongst the younger colleagues.

Staying hydrated was also important for the participating seafarers, with several noting that they find that water helps them in avoiding fatigue. It should be noted, however, that in busier times the participants said that they struggle to find time to drink, and some have very limited access to water, being prohibited to drink in front of the passengers. This is particularly a problem in the summer, when greater levels of water are required to remain hydrated, but is also when the ferries are busiest with passengers. Similarly, food was cited as an effective countermeasure to fatigue, but the point was made that breaks may be too short to provide sufficient time to eat anything substantial, particularly food which needs heating up.

Fresh air was cited as an effective countermeasure, with some participants going up on the deck of the vessel for a walk or to smoke. This is likely to be as part of a short informal break, which was also considered to be of use in avoiding fatigue. Such a break might also incorporate a short nap, which is an additional countermeasure used by participants. It should be noted here, though, that comments were made suggesting that a nap carries with it a danger of oversleeping.

Another view of feeling fatigued whilst at work was that there were no countermeasures that could work, and it was necessary to just continue working. For example, one participant said they would

“*just carry on. Just get on with it.”*

Another said they would endeavor to

“*work in energy saving mode”* and a third said they would “*try and keep busy”*.

Other participants said they did not use countermeasures because they were kept so busy working that they did not have the time to do so.

#### 3.4.1 Relaxing (nine focus groups)

Relaxing at the end of a shift was seen as a very important measure for combatting fatigue, particularly for those living on board the vessel. There was inevitably a range of methods of relaxing, including attending the gym and other sports, watching the TV, reading, and playing computer games. Those participants living on board were away from their family for extended periods. They therefore relied on online communication with home and so noted the importance of an effective internet connection. There was a feeling of isolation associated with not being able to connect with family and friends.

Importantly, many of the participating seafarers, whether living on-board or going home at the end of the shift noted the importance of having time to relax before going to bed to sleep. This was summed up by the participant who said:

“*I take quite a while to unwind … when I get home, I'll have a shower, get ready for bed and everything, and I have to kind of sit there, either watch a bit of telly or go on my phone … because if I try and go to sleep straightaway, it doesn't work for me”*.

### 3.5 Interaction between themes

The different elements considered across themes and sub-themes do not happen in isolation from each other. They interact to influence the experience of fatigue. For example, the external factor of bad weather has an impact on both physical fatigue (increasing physical effort of standing) and sleep related fatigue (reducing restful conditions for sleep). Bad weather may also increase shift duration if a journey is delayed, and the knock on impact to a person returning home to sleep is reduced time off between shifts, this is particularly impactful for a person with a long commute, as opportunity for sleep is already reduced compared to an individual with a short commute time.

Responses to the idea of reporting feeling fatigue varied between participants. One influencing factor here is workplace culture however, even within an open work place culture response to topics around fatigue may vary. For example, there was clear evidence of a seafaring community where those on a vessel were considered to be like family, including belief in being sufficiently close to be able to recognize fatigue in others. However, it was also suggested that an individual could be laughed at for reporting fatigue, suggesting that some topics are not open even within a close working group. There was also evidence that a workplace culture may be willing to increase fatigue risk in order to manage other safety risk, for example, the need to safely man the ship was recognized as highly important, and presented a reason why an individual might return to work if requested to do so at the expense of rest time. The different weighting applied to different safety related risks was apparent in terms of training, whereby drills are prioritized at the expense of sleep and rest (particularly for those off shift during a particular drill) but training for how to manage fatigue was rare for participants.

A complexity for the Ro-Pax industry is the necessity of both shifts and rosters to deliver the service. For example, some shifts may be sustainable at manageable fatigue levels for short rosters, but the same shift over a long duration roster may become fatiguing.

## 4 Discussion

In common with all shift workers seafarers are at high risk of experiencing sleepiness which has implications for the health and wellbeing of the crew but also for the safety of passengers. For customer-facing staff safety risk is related in particular to emergency response as they will assume responsibility for passenger safety during an emergency. Typically emergencies are medical or fire related. Beyond the universal aspects of shiftwork which can impact fatigue there are several reasons why those in customer-facing roles might be at heightened risk. Firstly, customer-facing staff have high workload for a sustained period of time. Unlike other crew whose busiest periods are during port operations (Kahveci, 1999), customer-facing staff attend to passengers for most of the voyage allowing them little opportunity for recovering from fatigue during shift. For example, some participants in the current study worked long 13-hour shifts with three unpaid 20min breaks during the shift giving little time away from customers. Dealing with passengers is one of the main stressors experienced by study participants and customer-facing crew have the responsibility of ensuring customer satisfaction during the crossing (Sonnentag and Zijlstra, 2006). Additionally, the hierarchy of seafaring operations allocates service staff in customer facing roles as lower ranking. For example, for staff in live-on-board operations, sleeping facilities may be less supportive of restful sleep than for more senior officers. Focus group participants reported sharing a cabin as a factor which disturbed their sleep, in addition to aspects such as general noise, vibration, weather and Tannoy systems.

Fatigue is a complex workplace health and safety issue and is contributed to by multiple factors. Causal factors of fatigue can originate from both the activity being undertaken (task related fatigue) and the physiological aspects of sleepiness. May and Baldwin (2009) explain this in their model related to driver fatigue as having three components: active task related fatigue (overload), passive task related fatigue (under load) and sleep related fatigue. For those working in seafaring customer facing roles, active task related fatigue, including physical fatigue from standing and dealing with customers, and sleep related fatigue, due to shift patterns and influences on sleep opportunity such as willingness for overtime and commuting, appear to have greater influence than passive task related fatigue. This is a different experience from other job roles where passive task related fatigue may be a concern e.g., for those with watch duty responsibilities. Being the public face of the company at sea, customer-facing crew are required to uphold the company's reputation (Abd-El-Salam et al., 2013). This high level of stress and responsibility can lead to fatigue (Jiandong et al., 2022). It may be that the shorter opportunity for lower activity within the job role and fewer breaks could lead to increased fatigue in this job role. There has been little previous research in fatigue experiences of customer-facing crew, and what is available is based on research outside of the seafaring environment (Dohrmann and Leppin, 2017).

There are obvious safety implications for a person falling asleep while in a position of responsibility, however, the implications for seafarers in customer facing roles are more nuanced. For example, sleep deprivation alters a person's engagement in social interactions to favor their own interests over fairness (Anderson and Dickinson, 2010) and increases impulsivity to negative stimuli (Anderson and Platten, 2011), this could have serious consequences in an emergency situation where the safety of an individual is at stake alongside the people they are responsible for. Sleepiness also impacts emotion and mood (Palmer et al., 2024) which has potential negative implications for customer experience. Despite the potential for impact, the focus group participants suggested that fatigue was not an issue during emergencies as adrenaline carries them through in these situations. It was the fatigue experienced after an incident which was of greater concern to them. Findings from this work evidence the preparation that goes into emergency planning, since it was not a concern to participants that they might be too tired to attend to an emergency situation safely. Multiple examples were provided where customers could be safely attended to despite the clear evidence of fatigue experienced at work. It is possible that the drill-based approach to safety within the maritime industry facilitates automatic behavior during an emergency, even when fatigued.

Sleeping on a vessel can lead to disturbed sleep (Hystad and Eid, 2016) and consequently insufficient sleep. For example, noise and vibration have been identified as stressors to seafarers (Lützhöft et al., 2010; Oldenburg et al., 2009; Wadsworth et al., 2006; Allen et al., 2008; Ellis et al., 2003), these factors were evident in these focus groups and perceived as being worse than for other job roles because of cabin location. Additionally, for those who must stand all shift in customer facing roles the vibration experience contributed to physical fatigue. Participants reported this experience to vary between vessels, suggesting that there is opportunity to mitigate this cause of fatigue on more modern ships, however, there are financial implications for updating the fleet. Furthermore, there is the issue of the movement of the vessel leading to motion sickness (Dobie, 2019), particularly during rough weather or storms that the seafarers are not accustomed to. Arguably those most prone to sea sickness are not likely to seek employment onboard a vessel, therefore it may be expected that all participants had some tolerance to seasickness. Interestingly, the physical environment was reported by some services staff as having differing impact on fatigue and seasickness, particularly in relation to certain vessels; it is notable that the variability reported between vessels suggests that ferry design could be optimized to mitigate the risk of fatigue. Repeated experience of disturbed sleep leads to both short-term fatigue and longer-term implications e.g., potential burnout (Ekstedt et al., 2006). During a work shift following sleep disturbance there will be greater risk of fatigue as sleep pressure will not have been sufficiently reduced (Ma et al., 2022).

Some of the focus group participants noted having had difficulty relaxing after work and on the vessel; this has been found to link with work-related stress in other fields such as healthcare where the link between stress, relaxation and sleep have been widely studied (Epstein et al., 2019; Gillet et al., 2020). Poor relaxation behaviors and difficulty sleeping have also been linked in non-seafaring populations (Jakobsson et al., 2020; Hedin et al., 2020). Although it is difficult to implement relaxation techniques outside of work, it may be beneficial to promote relaxation techniques to aid sleep during work time as this could embed techniques which may be used at home. Within seafaring, an empirical study by Allmer (1996), observed that focusing on coping strategies on board including a variety of passive recreation techniques such as calming down and settling down, relaxing, sleeping, listening to music, or watching DVDs is beneficial. It could be argued that Allmer (1996) research may be outdated as modern seafaring conditions have improved and more recent research has found that power napping may also be a helpful short term relaxation technique (Jensen and Oldenburg, 2020), which would also have benefits for counteracting insufficient sleep. Short naps were reported by participants in the focus groups so could be a possible strategy for fatigue management, however, passive recreation techniques were not.

Alongside difficulty relaxing at work, the difficulties of relaxing at home were an issue for the participating seafarers; those who are restless at home also have increased likelihood of fighting sleepiness at work. Returning home should be an opportunity to rest and reset, particularly when a seafarer is rostered onboard. If there are also significant life stressors outside of work which make it difficult to relax, then a seafarer's sleep routine could be compromised (Geiger-Brown et al., 2011). For seafarers who sleep on board there is a higher probability that they may miss important family and social events when at sea which could become a life stressor outside of work; this was a problem for the focus group participants. Recognizing the interaction between general health, stress and fatigue is necessary for inclusive fatigue management.

Although the focus group participants had their own ways of managing fatigue, they also reported that they had received little or no training on this issue, so for the most part these were likely to be informal solutions which they had arrived at themselves through personal experience and not those they were formally advised to use by their employer. Participants in the focus groups were likely to report countermeasures which could quickly be achieved often in their work location, such as taking caffeine, talking to a colleague, drinking water, or carrying on with reduced performance. These are in line with experiences in the rail (Filtness and Naweed, 2017) and bus industry (Miller et al., 2020), although a preference for caffeine was notably high in seafarers.

Napping was also mentioned by the focus group participants, although they did so in the context of relaxing rather than seeking sleep, possibly due to relatively shorter break opportunities. Generally napping has been shown to be an effective countermeasure to sleepiness (Pilkington-Cheney, 2023). Within some aviation operations, controlled napping has been shown to be beneficial (Hartzler, 2014), although there are organizational (“not seen as correct”) and operational (no facility to sleep) reasons why this might not be suitable.

Participants were able to recognize the experience of fatigue within themselves. The ability to recognize fatigue in oneself means that there is potential for a mitigation strategy to be put in place prior to elevated safety risk occurring. However, there was a mixed experience as to whether fatigue would be reported or not. One mechanism by which formal reporting of fatigue could be achieved is through implementing a fatigue risk management system (FRMS) (Williamson and Friswell, 2013). This is important because if employees do not report when fatigue is occurring employers do not know what risk they are facing. Feeling comfortable to report fatigue will only happen with an open safety culture.

Given the findings from the current work the following recommendations for those seeking to minimize fatigue in seafarers are made:

- Develop fatigue risk management programmes. This would increase the focus on fatigue, highlight that it is a serious issue and promote discussion.
- Invoke an open culture whereby fatigue is a topic which can be discussed.
- Provide education consistently across the ferry sector on fatigue management for all.
- Seek to limit the number of extra workdays and overtime seafarers can undertake, whilst recognizing this might affect seafarers earning potential and lead to alternative approaches to meet safe vessel manning.
- Consider options for two full crews to avoid the need to wake (e.g. for drills) a crew member who is sleeping while on rest or to delay the start of an off-duty period.
- Support seafarers' in access to communication with their significant social group to ensure they remain connected, as an approach to mitigating the stress experienced from work.
- Support seafarers health and wellbeing following response to an emergency.
- Seek to support fatigue management in onboard services staff and minimize disparity between experiences of this group and other job roles, for example, rest facilities and after care in the aftermath of incidents.
- Encourage development of a culture which encourages and normalizes reporting of fatigue and enables its impact to be monitored.
- Incentivise operators to invest in employee rest facilities, seeking options to minimize the impact of noise, vibration, other staff, and vessel operations on sleep opportunity.
- Modernize the ferry fleet.
- Avoid use of split shifts.
- Encourage consideration of fatigue risk in shift and roster planning.

## 5 Limitations

This research is the first of its kind to examine fatigue and its consequences for customer-facing seafarers. Nevertheless, and in common with most other research, this project experienced some limitations, partly due to the complexity of the ferry industry and the realities of field research. The specific focus of one job role provides in-depth insight into lived experience, however this means that there is no opportunity to triangulate data with other sources, such as management perspectives. A field study undertaken as part of the wider project, with which the current work is associated, identified that customer-facing staff report highest workload and relatively high subjective sleepiness compared to other job roles (Dahlman et al., 2026).

The target population of the focus groups was keen to participate and have their voices heard, and there was no evidence that they were unwilling to speak honestly about their experiences. However, there were logistical challenges. It is essential to have the support of the ferry operator to overcome the challenges of recruiting from this population and facilitating them being able to participate. It is possible that those operators who were most engaged in the research are also those who are already the most committed to managing fatigue. This means that the results may underrepresent the challenges of fatigue management for customer-facing seafarers. It should be noted, however, that attempts were made by the research team to include participants from as wide a range of operators as possible by advertising the focus group opportunity via sources additional to the operating companies. Additionally, it was not possible to undertake member checking of the analysis. The researchers undertaking the analysis were present during the data collection meaning that they were close to the data before analysis started, however, it is a limitation that it was not possible to confirm with participants that the findings are an accurate reflection of their experience.

It is noted that a bosun was inadvertently included in one of the focus groups (his role was not stated until a good way through the discussion); such a person might be considered to be the manager of the remaining ABs taking part in the discussion. In order to mitigate any effects, the researcher leading the group was careful to ensure that the ABs were comfortable with expressing their honest opinions in the presence of the bosun.

Ferry operators in the UK draw their employees from many countries and since the focus groups were conducted in English this may have had the effect of limiting participation. There may also be cultural factors which can influence the likelihood of participation or willingness to make negative comments; this may also have had an effect on the focus group population.

## 6 Conclusion

Understanding the lived experience of shift workers is an important first step to implementing workplace mitigation strategies to effectively reduce sleepiness related risks (Filtness et al., 2025). This study is one of the first to consider the experiences of fatigue of customer-facing seafarers.

Widespread experience of fatigue at work was found amongst this group within the ferry industry, alongside limited training on how to manage or mitigate it and barriers to reporting. Fatigue was partly a consequence of the same issues which affect other seafarers: shift and roster patterns, commuting, and external factors such as onboard noise and inclement weather.

However, fatigue appears to be further increased for this particular group by their interactions with passengers: and by their responsibilities for passenger safety, in terms of training for emergencies, their muster list duties and the aftermath of incidents. The active nature of their work introduces further fatigue impacts, in contrast to the more traditional focus in seafaring of needing to mitigate fatigue during monotonous activities.

Most of the literature on seafarer fatigue has considered those workers who may contribute most to accident risk, such as masters and watchkeepers. Customer service staff are not safety critical in this way; they explicitly stated in this research that fatigue did not compromise their ability to act effectively in cases of emergency.

This study, however, has for the first time identified the personal fatigue impact from the twin challenges of seafaring life and the responsibilities of customer facing duties. This is an important first step toward mitigating sleep related risks for these important but hitherto largely overlooked workers.

## Data Availability

The datasets presented in this article are not readily available because it contains interviews with a selection of persons. Sometimes persons share sensitive information about working conditions. Requests to access the datasets should be directed to s.e.maynard@lboro.ac.uk.
