# Correction: Seafarer Fatigue: a qualitative study of customer-facing staff working on UK ferries

**DOI:** 10.3389/frsle.2026.1993629

**Published:** 2026-09-29

**Authors:** Frontiers Production Office

**Affiliations:** Frontiers Media SA, Lausanne, Switzerland

**Keywords:** customer-facing seafaring staff, fatigue, risk factors, self-devised countermeasures, sleepiness

Affiliation “2 Loughborough University Transport Safety Research Center (TSRC), Wokingham, United Kingdom” was erroneously assigned to author Adam Asmal. The correct affiliation for this author at the time of the research was “1 School of Design and Creative Arts, Loughborough University Transport Safety Research Center (TSRC), Loughborough University, Loughborough, United Kingdom,” with a present address of “TRL, Wokingham, United Kingdom.”

The original version of this article has been updated.

